# Desert mycobiome of Saudi Arabia is driven by vegetation patterns

**DOI:** 10.3897/mycokeys.130.176937

**Published:** 2026-03-19

**Authors:** Israel Mani, Vladimir Mikryukov, Saad Alkahtani, Leho Tedersoo

**Affiliations:** 1 Institute of Ecology and Earth Sciences, University of Tartu, 2 Liivi, 50409 Tartu, Estonia Department of Zoology, College of Science, King Saud University Riyadh Saudi Arabia; 2 Mycology and Microbiology Center, Institute of Technology, University of Tartu, Nooruse 1, 50411 Tartu, Estonia Institute of Ecology and Earth Sciences, University of Tartu Tartu Estonia; 3 Department of Zoology, College of Science, King Saud University, 11451 Riyadh, Saudi Arabia Mycology and Microbiology Center, Institute of Technology, University of Tartu Tartu Estonia

**Keywords:** Desert ecosystem, environmental factors, fungal community composition, soil fungi

## Abstract

Deserts are home to diverse microbial communities important in many ecological processes and strategies for responding to a changing climate. We recorded the biodiversity of soil-inhabiting fungi and their predictors in Saudi Arabia via metabarcoding. Alpha diversity of the fungal communities varied greatly, with high diversity in moist montane habitats and very low diversity in hyper-arid regions. The fungal community was dominated by members of the orders Pleosporales, Pezizales, Agaricales, Glomerales, and Sordariales, most of which represented saprotrophic guilds. Modelling analyses showed that soil pH, elevation, vegetation coverage, and vegetation indices substantially impact soil fungal richness and community composition. These patterns mirror global dryland trends, with low diversity and high evenness in hyper-arid sites but higher richness and ecological differentiation in montane and vegetated regions. Our results demonstrate that vegetation cover, edaphic conditions, and altitude jointly shape fungal diversity in Saudi Arabian soils, offering mechanistic insight into community assembly and predicting ecosystem responses to climate change in arid landscapes.

## Introduction

Soils play a central role in global biogeochemical cycles and ecosystem health, with biodiversity and functions shaped by complex environmental, chemical, and physical predictors (Chen et al. 2019; Street et al. 2020; Zhang et al. 2024). The loss of biodiversity in response to climate change and anthropogenic activities compromises essential soil functions and ecosystem services (Wagg et al. 2014), significantly impacting microbes vital for soil formation, nutrient cycling, and ecosystem stability (Wang et al. 2017; Kang et al. 2021). This relationship is especially critical in arid and semi-arid regions, where species and genetic diversity underpin ecosystem resilience to environmental stressors (Zhang et al. 2023a). Drylands cover approximately 41% of Earth’s terrestrial surface, providing vital ecological functions despite being characterized by low rainfall, high evaporation, variable temperatures, and frequent drought events (Fu et al. 2021; Wang et al. 2022). Desert soils, generally sandy and deficient in organic matter and nutrients, are prone to erosion, drying, and high salinity and alkalinity. However, acidic niches may develop beneath the vegetative cover (Dwevedi et al. 2017; Naorem et al. 2023). Vegetation in desert regions is sparse, typically dominated by drought-adapted species (Oyedeji 2023; Wang et al. 2023; Tariq et al. 2024), yet these ecosystems sustain remarkable biodiversity and adaptation strategies under harsh conditions (Berdugo et al. 2022).

Recent studies highlight that desert soil microbes, particularly fungi, are crucial in supporting ecosystem services. Fungi significantly contribute to organic matter decomposition (Coban et al. 2022), nutrient cycling (Delgado-Baquerizo et al. 2020), carbon sequestration (Liu et al. 2020), and soil crust formation (Tian et al. 2021), thereby securing ecosystem persistence during extreme desiccation. Several fungal groups are adapted to water-poor conditions, withstanding desiccation by forming resistant spores or rehydrating their mycelia or lichen thalli. Certain fungal groups, including terricolous species, plant-associated fungi, hyphomycetes, yeasts, and micro colonial fungi, are adapted to arid climates (Murgia et al. 2019). In arid soils, fungi mediate nutrient and carbon cycling, interacting with plant communities to influence vegetation diversity and carbon storage through litter decomposition and nutrient uptake (Islam et al. 2024), and they often establish mycorrhizal associations, enhancing plant drought tolerance (Bhantana et al. 2021). Additionally, fungi are integral in developing biocrusts, producing extracellular polysaccharides that improve soil stability, reduce erosion, and enhance moisture retention (Dhawi 2023). Despite their significant ecological roles, desert fungal communities, their functional attributes, and their adaptation mechanisms remain poorly understood. Thus, understanding fungal ecology represents an essential frontier for research to maintain dryland ecosystem sustainability under increasing environmental stress (Coleine et al. 2024; Mousa et al. 2024).

Saudi Arabia occupies approximately 80% of the Arabian Peninsula, encompassing diverse ecosystems such as montane grasslands, dry savannas, deserts, and coastal wetlands (Albaqami et al. 2018; Baeshen et al. 2023). The region’s climate is predominantly arid to semi-arid, characterized by low precipitation, high evaporation rates, high temperatures, and periodic drought over much of the year (Almazroui 2020; Alrajhi et al. 2024). Despite the ecological uniqueness and extensive geographic coverage, research on fungal communities within Saudi Arabia remains in its early stages. Arbuscular and ectomycorrhizal fungi have been documented to enhance native plant survival in harsh environments, improving plant resistance to drought, salinity, and heavy metals (Al-Ghamdi and Jais 2012; Albaqami et al. 2018). Recent molecular and metagenomic studies have detected previously unreported fungal taxa in diverse habitats including sand dunes, date palm plantations, and biological soil crusts, indicating substantial undocumented fungal diversity across the region (Baeshen et al. 2014; Kazeeroni and Al-Sadi 2016; Murgia et al. 2019; Symanczik et al. 2020). Available surveys suggest that Saudi Arabian soil mycobiota is dominated by Ascomycota, with Eurotiomycetes and Dothideomycetes being particularly common, and xerotolerant genera such as *Aspergillus*, *Alternaria*, and *Penicillium* frequently reported (Ameen et al. 2021). These taxa contribute to organic matter turnover and nutrient cycling under high temperature and salinity stress (Al Tamie 2014; Hashem et al. 2016; Murgia et al. 2019). Fungal community composition also varies among microhabitats and substrates (e.g., bare soils, biological crusts, dust deposits, and plant rhizospheres), reflecting environmental heterogeneity and wind-mediated dispersal (Abed et al. 2019; Ameen et al. 2021). However, systematic research is still required to elucidate the ecological roles, diversity, and adaptation mechanisms of fungal communities in the Arabian Peninsula.

This study aims to investigate soil fungal diversity across various habitats of Saudi Arabia. We hypothesized that soil fungal communities exhibit significant taxonomic and functional turnover across distinct environmental gradients. We predicted that environmental variables such as soil pH, elevation, vegetation type, coverage, and nutrient availability shape fungal diversity patterns and community composition through niche filtering. Here, we combined high-throughput sequencing of fungal communities with extensive environmental characterization, offering far-reaching insights into the diversity, composition, and environmental drivers of desert fungal diversity.

## Materials and methods

### Site description and sampling

The Saudi Arabian sites were distributed geographically from 16°N to 29°N latitude and 34°E to 50°E longitude, covering much of the country and capturing a wide array of environmental conditions. These ecosystems include sandy and volcanic deserts, sparse grasslands, riparian savannas, and suburban habitats. Generally, Saudi Arabia has a desert climate, except for its semi-arid southwest region and tallest mountain peaks, which are more humid. Summer temperatures range from 27 °C to 43 °C inland and from 27 °C to 38 °C near the coast. Winter temperatures are between 8 °C and 20 °C inland and 19 °C and 29 °C along the Red Sea coast. There is an annual rainfall of less than 150 mm, apart from the southwestern region, which receives up to 600 mm.

Soil samples were collected from 85 locations throughout Saudi Arabia in winter from 2020 to 2024 (Suppl. material 1: Fig. S1). Sampling was performed using the approach described by Tedersoo et al. (2021). At each site (56-m diameter circular plot), 40 subsamples with a diameter of 5 cm to a depth of 5 cm were collected and subsequently pooled. This 0–5 cm sampling depth follows the Global Soil Mycobiome protocol and targets the biologically active surface layer; in desert soils, it also reduces dilution by low-biomass mineral subsoils, improving comparability across studies (Tedersoo et al. 2021). Composite samples were air-dried at room temperature and homogenised by hand, followed by bead beating. Two grams of soil from each sample were used for molecular analysis. The remaining soil sample was further dried and used for physicochemical assessment. Vegetation cover was estimated at each site as the percentage (0–100%) of total canopy within the plot. Vegetation age was categorized into three classes (young, mature, or mixed) based on the predominant successional stage and the size of the class of perennial species within each plot. Biome types were classified according to the RESOLVE ecoregion classification system (Dinerstein et al. 2017), which categorizes habitats based on regional climatic and botanical characteristics as determined by the Global Soil Mycobiome consortium (Tedersoo et al. 2021).

### Molecular assessment

The DNA extraction, amplification, and sequencing protocols followed the methods outlined in Tedersoo et al. (2021). Environmental DNA was extracted using the MagAttract PowerSoil Kit (Qiagen GmbH, Hilden, Germany) from homogenised soil samples in accordance with the manufacturer’s instructions. To improve taxonomic resolution and accuracy, a long-read sequencing approach was used, targeting the internal transcribed spacer (ITS) region. PCR amplification was performed with indexed, eukaryote-specific primers ITS9mun (GTACACACCGCCCGTCG) and ITS4ngsUni (CGCCTSCSCTTANTDATATGC). The PCRs were carried out in duplicate in 25 μl reactions using 5 × HOT FIREPol® Blend Master Mix (Solis BioDyne, Tartu, Estonia). The PCR mixture consisted of 5 μl of HOT FIREPol Blend Master Mix, 0.5 μl of forward and reverse primers, 1 μl of DNA extract, and 18 μl of ddH_2_O. Thermal cycling comprised an initial denaturation at 95 °C for 15 minutes, followed by 25 cycles of denaturation at 95 °C, annealing at 57 °C, and extension at 72 °C, with a final elongation at 72 °C for 10 minutes. The samples were further stored at 4 °C. Reamplification was performed on samples that did not yield visible amplicons, using either 28 or 30 cycles. A library pool was formed by adding 1–10 μl of PCR product to the pool of amplicons, based on the intensity of the amplicon band on a gel. Using the FavorPrep^TM^ GEL/PCR Purification Kit (Favorgen, Ping-Tung, Taiwan), the pooled amplicons were purified and then shipped to the University of Oslo Sequencing Centre for PacBio library preparation and sequencing. Sequencing was carried out using PacBio’s Single Molecule Real-Time (SMRT) technology (Pacific Biosciences, Palo Alto, USA) on the Sequel II platform with a SMRT Cell 8M, and CCS (Circular Consensus Sequencing) reads were generated for further analysis (Tedersoo et al. 2022).

### Bioinformatic assessment

Bioinformatic analysis was performed using the NextITS pipeline v.1.0.0 (Mikryukov et al. 2025). Demultiplexing relied on LIMA v.2.12.0 (Pacific Biosciences) with dual 12-bp indices and a minimum barcode score of 93, and any read lacking both primer sites was discarded after adapter and primer trimming with cutadapt v.5.0 (Martin 2011). Full-length internal transcribed spacer (ITS) regions were then retrieved by ITSx v.1.1.3 (Bengtsson‐Palme et al. 2013), and sequences shorter than 250 bp, or containing more than 0.6 expected errors per 100 bp, or harbouring homopolymers exceeding 25 nt were removed. Chimeras were detected in a two-step scheme: an initial *de novo* UCHIME screen with VSEARCH v.2.29.4 (Rognes et al. 2016) with a maximum chimera score of 0.6 (Nilsson et al. 2015), followed by reference-based verification against the EUKARYOME v.1.9.4 (Tedersoo et al. 2024) database, with any sequence flagged in either step being excluded. Surviving reads were clustered at 98% pairwise similarity with VSEARCH. Steps of the bioinformatic analysis were orchestrated using Nextflow v.25.04.6 (Di Tommaso et al. 2017) workflow manager. Representative sequences of each operational taxonomic unit (OTU; Blaxter et al. 2005) were queried with BLASTn v.2.16.0+ against the EUKARYOME database, retaining the ten best hits. All kingdom-level-unclassified OTUs and non-target amplicons (e.g., bacterial) were removed. Fungal species were identified by matching them with the UNITE species hypotheses (SHs) (Abarenkov et al. 2022). Ecological guilds and trophic modes were annotated by cross-referencing with the FungalTraits database v.1.2 (Põlme et al. 2021) and treated as inferred ecological roles, acknowledging incomplete trait coverage for arid-soil fungi. Krona charts were used to illustrate the hierarchical taxonomic distribution (Ondov et al. 2011).

### Soil parameters and environmental variables

In accordance with the procedure detailed by Tedersoo et al. (2012), 20 g of dried, homogenized material was used to evaluate the physicochemical characteristics of the composite soil samples. Soil pH (in KCl), potassium (K), magnesium (Mg), calcium (Ca), phosphate (P), total organic carbon (TOC), and total nitrogen (TN) were measured. Furthermore, the δ^15^N and δ^13^C isotope abundances were quantified. The C:N ratio was determined by dividing the TOC content by the TN concentration in the soil. The bioclimatic variables, with a 30 arcsecond resolution (about 1 km), were obtained as GeoTIFF images from the WorldClim v2.1 dataset (Fick and Hijmans 2017). These variables are CMIP6 projections from the MIROC6 Global Climate Model (GCM) under the Shared Socio-economic Pathways (SSP245), representing near-term climatic conditions (2021–2040). We used this near-term layer as a proxy for contemporary climate during the sampling period, given that the widely used WorldClim “current” baseline (1970–2000) predates recent warming trends in the region. We utilized Google Earth Engine (GEE; https://earthengine.google.com) (Gorelick et al. 2017) to generate annual composites of four significant vegetation and water indices across 85 locations in Saudi Arabia from the years 2020 to 2024. These indices are the Normalized Difference Vegetation Index (NDVI), the Normalized Difference Water Index (NDWI), the Enhanced Vegetation Index (EVI), and the Land Surface Water Index (LSWI). The NDVI and NDWI were computed using the MODIS MOD09A1 data (Vermote 2015) (8-day surface reflectance at 500 m resolution), following the approach provided by Gu et al. (2008), which is commonly used for vegetation and drought monitoring. The EVI and LSWI indices were calculated using MOD13Q1 data (Didan 2015), which provides vegetation information every 16 days at a 250 m resolution, based on the land cover classification method described by Kou et al. (2017). An annual composite was generated using the maximum value composite (MVC) technique inside the GEE environment, after quality assurance flags were used to remove pixels tainted by clouds. Utilizing this approach allowed for high-quality time series analysis to analyze changes in vegetation, surface water supply, and weather over a five-year period.

### Statistical assessment

All statistical analyses were conducted in R v.4.4.1 (R Core Team 2024). To stabilize variance and approximate normality, environmental variables were log- or square-root-transformed prior to statistical analysis. Alpha diversity was measured by estimating OTU richness, Shannon diversity index (H'), and Pielou’s evenness (J') using the vegan package v.2.7-2 in R (Oksanen et al. 2025). To account for variation in sequencing depth across samples, a linear regression of log-transformed OTU richness against log-transformed sequencing depth was performed using the lm function, and the resulting residuals were used as sequencing-depth-corrected richness values (Tedersoo et al. 2022). The Shannon diversity index was calculated with the diversity function (index = “shannon”), and Pielou’s evenness was calculated as J' = H' / ln(S), where S is OTU richness. Before beta-diversity analyses, the OTU table was rarefied to 1,000 sequences per sample using the rrarefy function (vegan package) to account for unequal sequencing depth. We then computed Bray-Curtis dissimilarities using the rarefied data and performed permutational multivariate analysis of variance (PERMANOVA; Anderson 2001) with the adonis2 function. We used non-metric multidimensional scaling (NMDS; metaMDS function) on fungal OTUs to assess similarities and differences in community composition among soil samples. The envfit function fitted environmental factors onto the ordination, showing their association with fungal community structure. The analysis tested the influence of various environmental factors on fungal community structure with 999 permutations to determine the significance of these effects. Both the taxonomic and functional characteristics of the fungal community were examined. Pearson correlation and Mantel tests were performed using the vegan package to determine linear correlations and distances between the fungal community and environmental variables. Heat maps were generated using the pheatmap package v.1.0.13 (Kolde 2025) to visualize these relationships. We evaluated the effects of environmental predictors on fungal alpha diversity after screening for multicollinearity using variance inflation factors (VIF) with the car package v.3.1.3 (Fox and Weisberg 2019) and removing predictors with VIF > 5. OTU richness and Shannon diversity were modelled with generalized linear models (Gaussian family), with model selection by stepwise AIC (stepAIC function, MASS package v.7.3.65 (Venables and Ripley 2002)). Pielou’s evenness, which ranges from 0 to 1, was modelled using beta regression (betareg package v.3.2.4, Kosmidis and Zeileis 2025), and model selection was performed using MuMIn package v.1.48.11 (Bartoń 2025).

## Results

### Taxonomic composition and functional guilds of desert soil fungi

**Figure 1.**
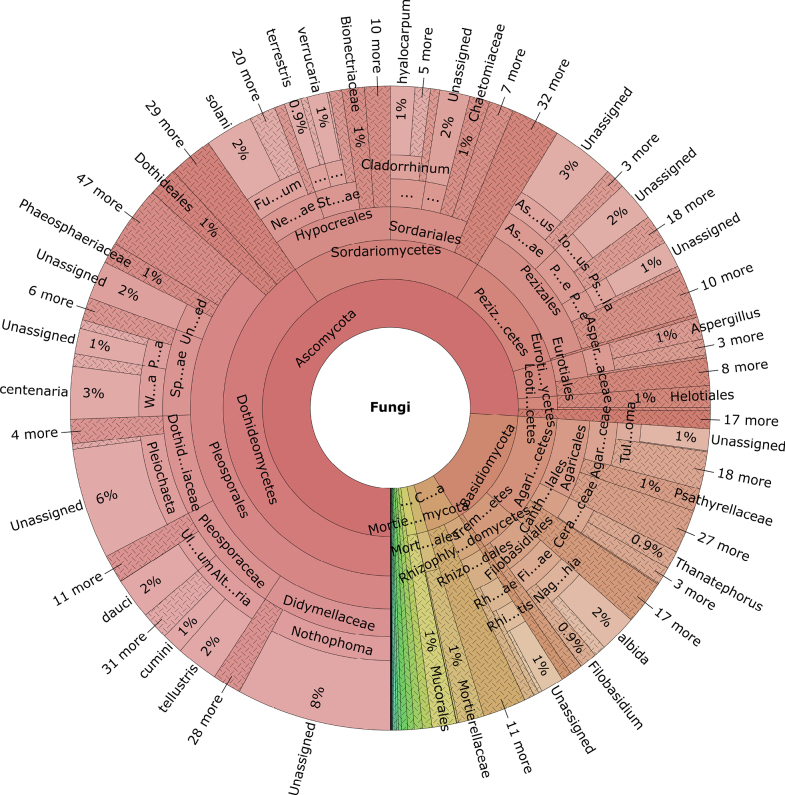
Taxonomic composition and relative abundance of fungal communities in Saudi Arabian soils, displaying their hierarchical distribution from the phylum to the genus level. (https://github.com/Mycology-Microbiology-Center/Desert-mycobiome-of-Saudi-Arabia-/blob/main/fungal_krona.html).

After quality filtering and clustering at 98% sequence similarity, our dataset consisted of 1,076,877 high-quality reads ascribed to 43,333 OTUs (Fig. 1). At the kingdom level, Fungi dominated (731,883 reads; 14,634 OTUs), representing 68.0% of the total reads, followed by Alveolata (156,500 reads; 10,743 OTUs), Viridiplantae (56,750 reads; 1,453 OTUs), Metazoa (49,392 reads; 4,605 OTUs), Amoebozoa (24,483 reads; 4,300 OTUs), Straminipila (14,247 reads; 1,740 OTUs), and Rhizaria (13,966 reads; 2,683 OTUs). Within Fungi, Ascomycota harboured 8,145 OTUs (76.3% of total fungal reads), followed by Basidiomycota (2,104 OTUs; 14.8% of total fungal reads), Chytridiomycota (1,127 OTUs; 4.3% of total fungal reads), Mortierellomycota (312 OTUs; 1.4% of total reads), and Mucoromycota (242 OTUs; 1.2% of total reads); the remaining 27 phyla each comprised < 0.5% of OTUs. Pleosporales was the most abundant order, comprising 2,568 OTUs (36.3% of total reads). Other prominent orders included Pezizales (1,194 OTUs; 12.7% of total reads), Hypocreales (746 OTUs; 9.3% of total reads), Agaricales (835 OTUs; 6.5% of total reads), Sordariales (665 OTUs; 6.2% of total reads), Filobasidiales (136 OTUs; 3.2% of total reads), and Rhizophlyctidales (285 OTUs; 2.1% of total reads). The remaining orders accounted for a combined total of 23.71%.

A total of 1,048 fungal genera were identified in the soil dataset. Among the annotated genera, *Alternaria* was the most diverse (179 OTUs, 4.4% of total reads), followed by *Ascobolus* (156 OTUs; 3.2%), *Pleiochaeta* (139 OTUs; 5.9%), *Westerdykella* (125 OTUs; 3.4%), *Naganishia* (82 OTUs; 2.4%), *Nothophoma* (72 OTUs; 7.8%), *Fusarium* (72 OTUs; 3.5%), *Cladorrhinum* (52 OTUs; 2.0%), and *Ulocladium* (46 OTUs; 2.0%). The five most common OTUs included *Alternaria* (80 sites; 5.4%), *Ascobolus* (78 sites; 3.7%), *Fusarium* (77 sites; 4.4%), *Nothophoma* (77 sites; 9.16%), and *Preussia* (75 sites; 2.0%). The most diverse sites were Bani Aamer (298 distinct genera; 28.4%), Maksanah (253 distinct genera; 24.1%), Almandaq (252 distinct genera; 24.0%), and Al-Malwi (231 distinct genera; 22.0%), predominantly in the southwestern highlands and northwestern escarpments (mountainous or vegetated habitats). In contrast, the least diverse sites were Thwaileil (7 distinct genera), Razor Bay (13), Al Khaldiyah (14), and Alwasel (17), primarily situated in hyper-arid desert or coastal environments with extreme dryness and sparse vegetation. The top 30 OTUs are given in Table 1.

**Table 1.** Site occupancy and relative abundance of the top 30 fungal species (as defined by UNITE species hypotheses) from ITS rDNA metabarcoding of composite topsoils across Saudi Arabia.

S.no	Fungal Species	SH	Frequency	Total relative abundance
1	*Nothophoma* sp.	SH0085547.10FU	77	5.2412
2	*Pleiochaeta* sp.	SH0085665.10FU	51	3.2323
3	*Westerdykella centenaria*	SH0175734.10FU	63	1.7675
4	*Fusarium solani*	SH0001363.10FU	68	1.5317
5	*Alternaria tellustris*	SH0081764.10FU	53	1.4313
6	*Ulocladium dauci*	SH0081549.10FU	57	1.3976
7	*Naganishia albida*	SH0157913.10FU	62	1.2630
8	*Cladorrhinum hyalocarpum*	SH0166229.10FU	43	0.8073
9	*Pseudombrophila* sp.	SH0123266.10FU	46	0.8064
10	*Myrothecium verrucaria*	SH0178204.10FU	52	0.6783
11	*Paramyrothecium terrestris*	SH0178319.10FU	39	0.5717
12	*Ascobolus denudatus*	SH0131262.10FU	41	0.5605
13	*Sporormia grandispora*	SH0037734.10FU	50	0.5039
14	*Rhizopus microsporus*	SH0168747.10FU	35	0.4223
15	*Penicillium polonicum*	SH0140458.10FU	50	0.4034
16	*Phaeomycocentrospora* sp.	SH0085595.10FU	30	0.3841
17	*Iodophanus carneus*	SH0116545.10FU	51	0.3759
18	*Fusariella atrovirens*	SH0132550.10FU	44	0.3554
19	*Cladosporium xylophilum*	SH0155802.10FU	60	0.3412
20	*Stromatinia* sp.	SH0145284.10FU	15	0.3199
21	Didymellaceae sp.	SH0085650.10FU	17	0.2897
22	*Lepidosphaeria nicotiae*	SH0037722.10FU	4	0.2831
23	*Pseudoarthrographis* sp.	SH0161317.10FU	16	0.2686
24	*Ceratobasidium* sp.	SH0152858.10FU	17	0.2565
25	*Trichobolus* sp.	SH0135003.10FU	10	0.2527
26	*Curvularia nicotiae*	SH0081706.10FU	53	0.2421
27	*Neodidymelliopsis polemonii*	SH0085547.10FU	39	0.2393
28	*Preussia terricola*	SH0188317.10FU	56	0.2211
29	*Aspergillus caespitosus*	SH0104476.10FU	24	0.2182
30	*Stagonosporopsis cucurbitacearum*	SH0085547.10FU	56	0.2148

**Figure 2.**
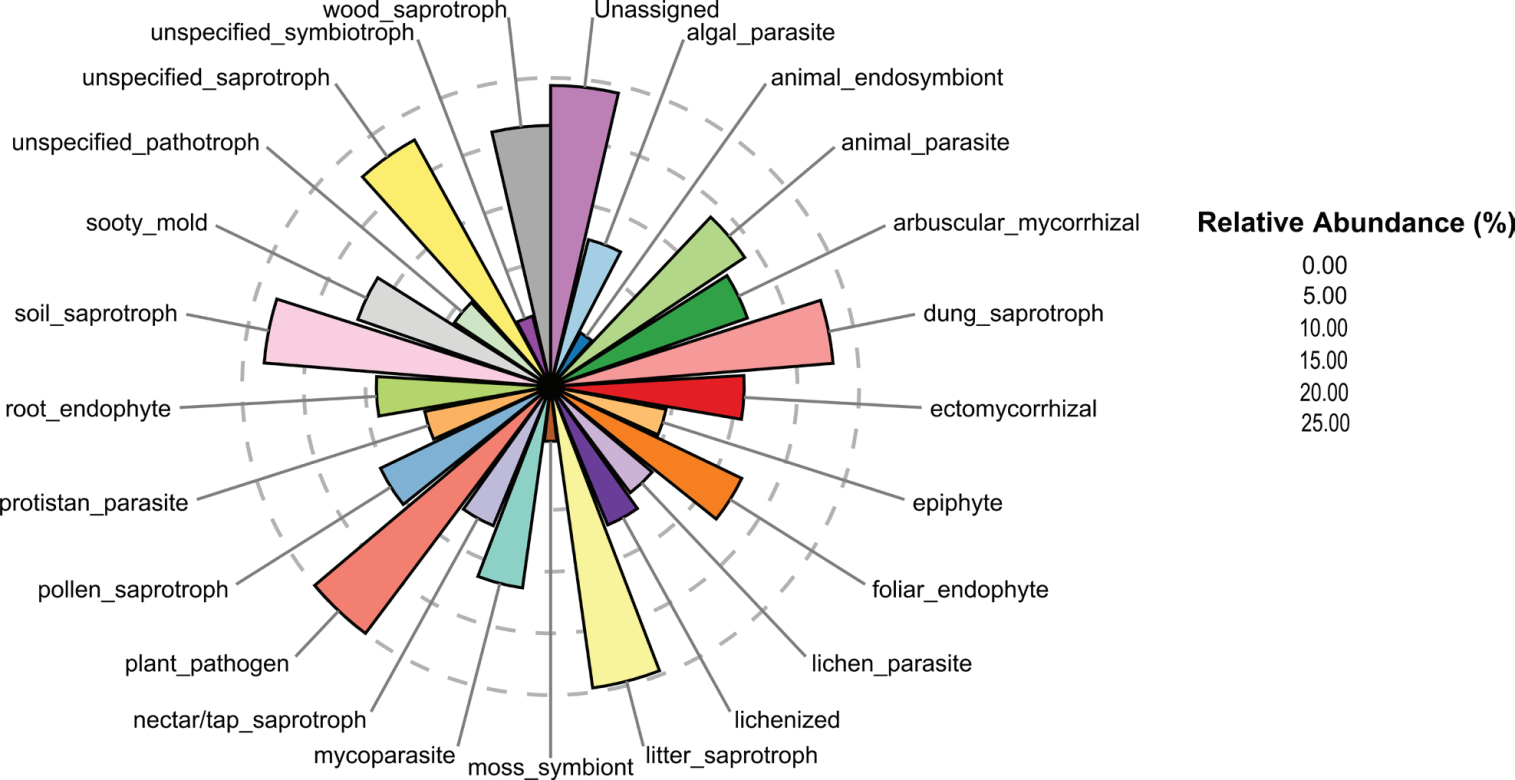
The diversity of fungal functional traits in Saudi Arabian desert soil samples, revealing significant variation in their composition and dominance among samples.

Using the FungalTraits database, we annotated primary fungal lifestyles at the genus level (Fig. 2). These assignments should be interpreted cautiously, as trait databases have incomplete coverage for arid-soil fungi and many desert lineages likely exhibit context-dependent lifestyles. Overall, saprotrophic fungi dominated the community at 53.2% of reads, including soil saprotrophs (10.7% of fungal reads; *Iodophanus* and *Paramyrothecium*), litter saprotrophs (21.1%; *Nothophoma* and *Ascobolus*), wood saprotrophs (3.7%; *Neocamarosporium* and *Thecotheus*), and dung saprotrophs (9.1%; *Westerdykella* and *Preussia*). Pathotrophs accounted for 27.0% of reads, dominated by plant pathogens (25.0%; *Pleiochaeta* and *Alternaria*), followed by animal parasites (1.2%; *Beauveria* and *Parengyodontium*), mycoparasites (0.36%; *Papiliotrema* and *Trichoderma*), lichen parasites (0.02%; *Monodictys* and *Heterocephalacria*), protistan parasites (0.02%; *Paramicrosporidium* and *Acaulopage*), algal parasites (0.04%; *Paraphelidium* and *Olpidium*), and sooty molds (0.37%; dominated by *Aureobasidium*). Symbiotrophs represented 1.4% of the community, mainly arbuscular mycorrhizal fungi (0.44%; *Dominikia* and *Rhizoglomus*), ectomycorrhizal fungi (0.24%; *Picoa*, potentially associating with the introduced *Eucalyptus* spp. and native *Helianthemum lippii*), root endophytes (0.11%; *Darksidea* and *Serendipita*), foliar endophytes (0.51%; *Bartalinia* and *Anthostomella*), epiphytes (0.01%; *Symmetrospora* and *Buckleyzyma*), and lichenized fungi (0.04%; *Arthopyrenia* and *Acarospora*), as well as minor groups such as animal endosymbionts (0.001%; *Tromeropsis*) and moss symbionts (0.001%; *Octospora*). Finally, 18.4% of fungal reads comprised OTUs with unresolved primary lifestyle traits.

### Distribution of soil fungal alpha diversity

Soil fungal diversity varied strongly across Saudi Arabian soils, ranging from 8 OTUs in the most hyper-arid deserts to 1,185 OTUs in moister areas, with a median value of 349 OTUs (IQR: 206–509). The Shannon diversity index (H') of the soil fungal communities ranged from 2.08 to 6.53 (mean ± SD: 5.44 ± 0.78), broadly mirroring the spatial pattern of OTU richness across the aridity gradient. Evenness values averaged 0.023 and ranged from 0.006 to 0.161.

Generalized linear models indicated that environmental factors collectively explained a substantial portion of the variation in fungal diversity (Suppl. material 1: table SS1). The final model for the OTU richness accounted for 49.5% of the variation, with the strongest statistical signals for vegetation coverage (positive effect, adjusted R^2^ = 0.226, *p* < 0.001) and soil pH (negative effect, adjusted R^2^ = 0.180, *p* = 0.007), and a positive association with elevation (adjusted R^2^ = 0.095, *p* < 0.001).

The final model for Shannon diversity explained 52.1% of the variation. Vegetation coverage (adjusted R^2^ = 0.178, *p* < 0.001) and elevation (adjusted R^2^ = 0.152, *p* < 0.001) were positively associated with H', whereas soil pH showed significant negative association (adjusted R^2^ = 0.173, *p* = 0.004).

Beta regression model showed that Pielou’s evenness was structured by a different set of factors, explaining 19.4% of the variation (pseudo-R^2^ = 0.194). Elevation (partial pseudo-R^2^ = 0.091, *p* = 0.021) and vegetation coverage (partial pseudo-R^2^ = 0.072, *p* = 0.006) both had significant negative effects. The vegetation index LSWI was the only significant positive predictor of evenness (partial pseudo-R^2^ = 0.050, *p* = 0.018). While soil nutrients such as calcium and potassium showed statistical significance in specific models (*p* < 0.05), they accounted for only a minimal proportion of the total variation (adjusted R^2^ < 0.05). This indicates a limited ecological signal and suggests that these nutrients are secondary determinants rather than primary drivers of fungal community structure in this system.

### Beta diversity and environmental drivers of soil fungal communities

The results of the PERMANOVA analysis indicated that multiple environmental variables had a substantial impact on the composition of the soil fungal community (Table 2). Vegetation indices (particularly NDWI) explained the largest portion of the overall variation, with additional contribution of elevation, soil pH, vegetation age, and soil nutrients (Table 2). Climatic predictors such as mean diurnal range, isothermality, and annual mean temperature further explained smaller but significant portions of variation. Surprisingly, fungal communities were unaffected by a wide range of other environmental variables like other soil nutrients (C:N ratio, total organic carbon, and total nitrogen) as well as most precipitation- and other temperature-related metrics.

**Table 2.** The impact of environmental parameters on fungal community composition based on PERMANOVA analysis.

Environmental variables	df	adj.R^2^	F	p value
NDWI	1.46	0.0317	4.956	0.001
Elevation	1.46	0.0230	3.968	0.001
Soil pH	1.46	0.0150	3.063	0.001
Vegetation age	1.46	0.0143	2.989	0.001
EVI	1.46	0.0119	2.715	0.001
Mean diurnal range	1.46	0.0096	2.452	0.001
Potassium	1.46	0.0084	2.316	0.001
Vegetation coverage	1.46	0.0082	2.291	0.002
Phosphate	1.46	0.0081	2.278	0.002
LSWI	1.46	0.0078	2.246	0.001
Isothermality	1.46	0.0060	2.039	0.001
Annual mean temperature	1.46	0.0031	1.6981	0.001

**Figure 3.**
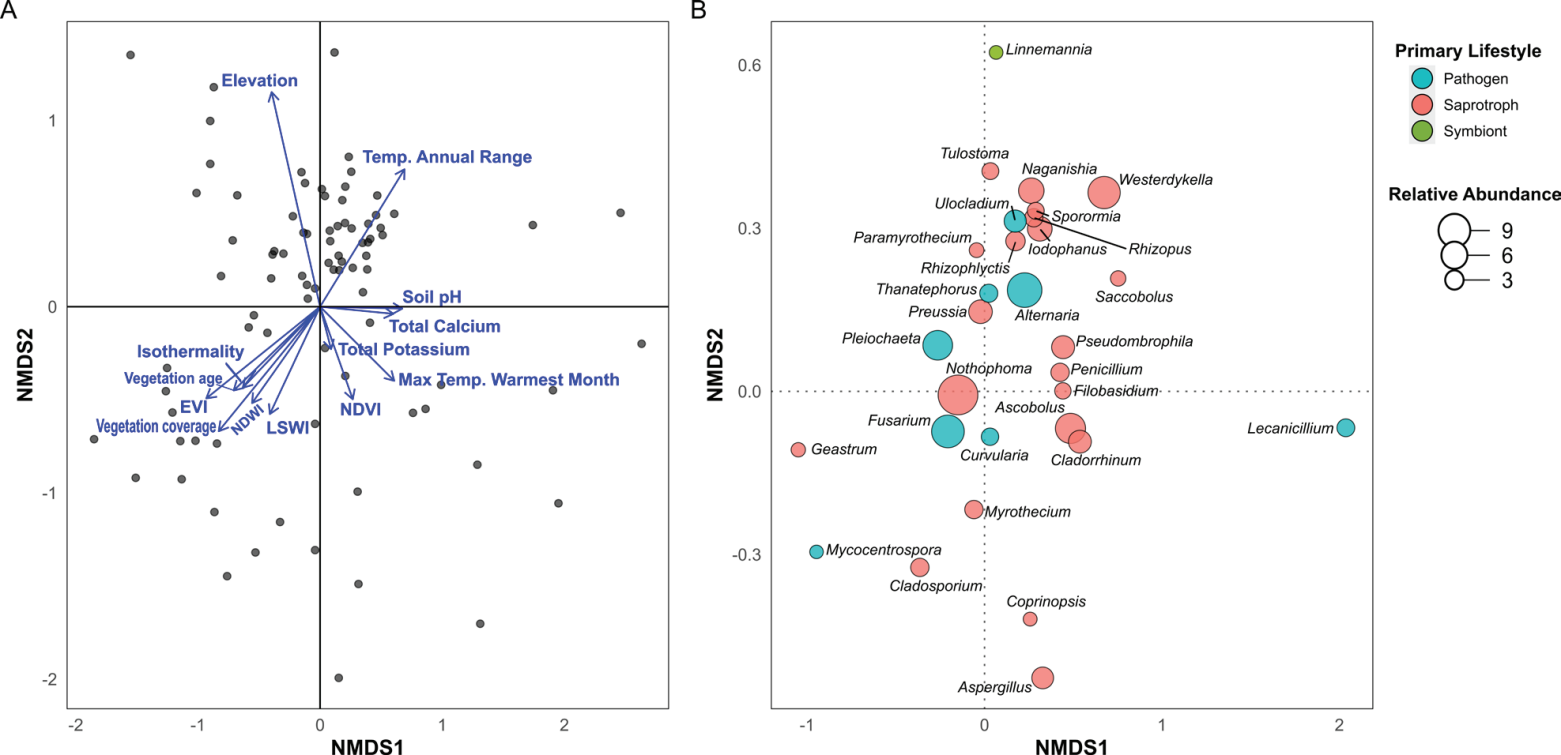
**A**. The NMDS ordination demonstrates the relationship between significant environmental gradients and variation in soil fungal communities, illustrating how multiple factors act together to structure diversity patterns across the study area; **B**. NMDS bubble plot of top fungal genera in Saudi Arabian soils, representing each genus with its primary ecological lifestyle and relative abundance.

The NMDS ordination illustrates clear structuring of fungal communities along major environmental gradients (Fig. 3A), with Axis 1 and Axis 2 explaining 48.2% and 30.6% of the variance, respectively, indicating that a substantial portion of the community structure was captured by two dimensions. The NMDS1 axis largely reflected edaphic variation (particularly soil pH and calcium, with R^2^ = 0.121 and R^2^ = 0.048, respectively), while NMDS2 was more strongly associated with topography and climatic factors (e.g., elevation and temperature range, R^2^ = 0.088 and R^2^ = 0.036, respectively). Productivity-related variables such as EVI (R^2^ = 0.054) and vegetation coverage (R^2^ = 0.054) clustered in the lower-left quadrant, while vegetation indices (NDVI, R^2^ = 0.023; LSWI, R^2^ = 0.022) and vegetation age (R^2^ = 0.020) were positioned orthogonally, suggesting partly distinct association with community turnover.

The NMDS ordination of the top 30 fungal genera (selected by total relative abundance across all samples; NMDS stress = 0.151; Fig. 3B) showed distinct patterns, with genera clustering by primary lifestyle. Genera such as *Naganishia*, *Pleiochaeta*, *Nothophoma*, and *Westerdykella* were positioned near the origin of both axes, indicating widespread occurrence and relatively even abundance across sites. In contrast, genera *Rhizopus*, *Thanatephorus*, *Preussia*, *Cladophialophora*, *Ascobolus*, and *Aspergillus* were located farther from the centre along one or both axes, suggesting more restricted distributions or stronger associations with specific conditions. These patterns highlight both generalist and specialist genera within the fungal communities of Saudi Arabian desert soils.

### Environmental determinants of soil fungal community structures

**Figure 4.**
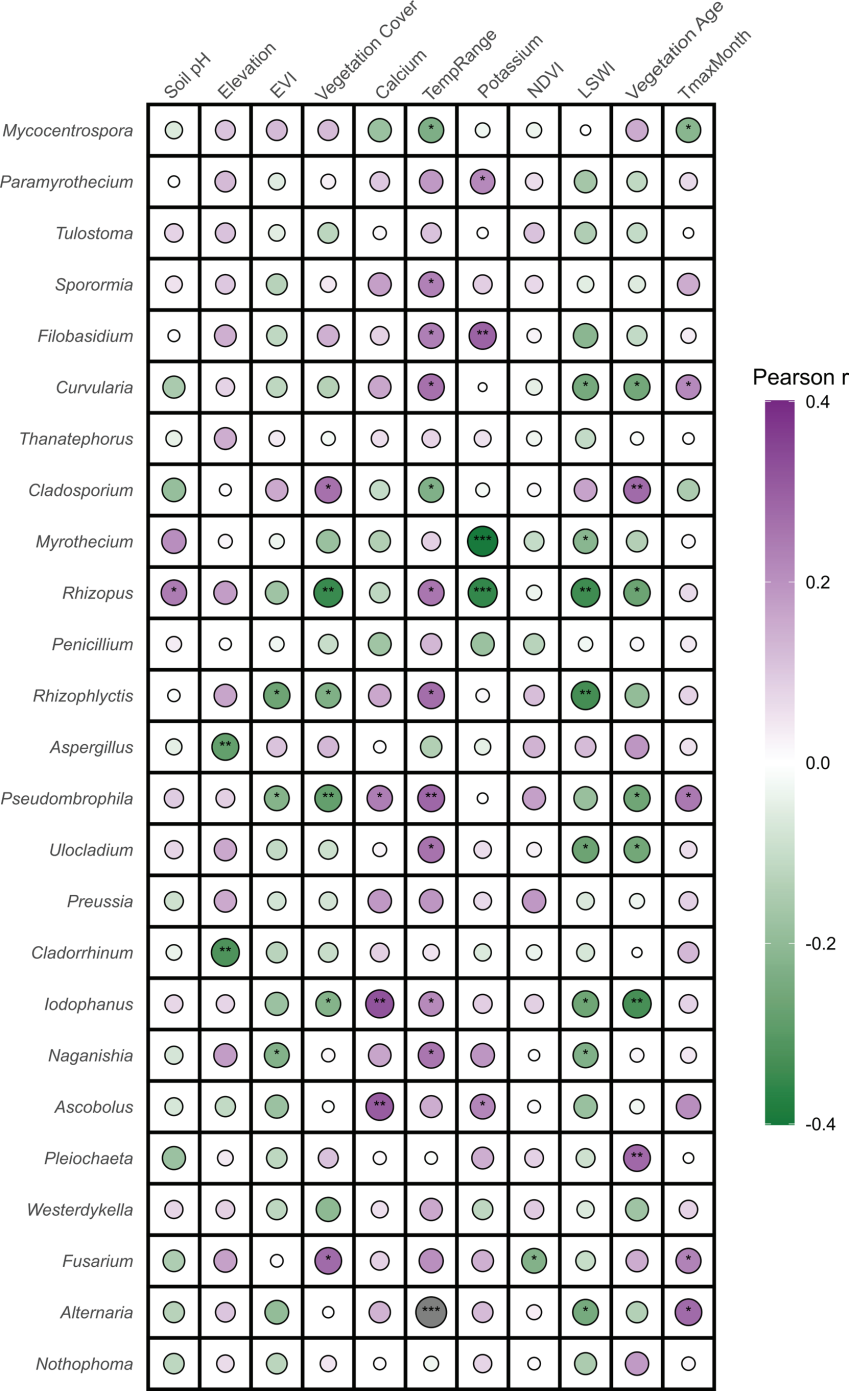
Correlogram showing Pearson correlations between the most frequently recorded fungal genera and environmental variables in Saudi Arabian soils.

The relationships between environmental variables and the relative abundance of dominant fungal genera were assessed using Pearson correlation analysis (Fig. 4). Significant correlations indicate genus-level associations with environmental gradients, whereas a few genera displayed no significant relationships, indicating complex or weak environmental responses. Temperature annual range was positively correlated with *Alternaria* (r^2^ = 0.177, p < 0.001), *Naganishia* (r^2^ = 0.065, p < 0.05), *Iodophanus* (r^2^ = 0.046, p < 0.05), *Rhizopus* (r^2^ = 0.065, p < 0.05), and *Curvularia* (r^2^ = 0.071, p < 0.05). Vegetation indices (LSWI, NDVI, EVI) and Vegetation cover were negatively correlated with *Alternaria* (r^2^ = 0.063, p < 0.05), *Naganishia* (r^2^ = 0.052, p < 0.05), *Myrothecium* (r^2^ = 0.046, p < 0.05), and *Rhizophlyctis* (r^2^ = 0.113, p < 0.01), while *Fusarium* (r^2^ = 0.077, p < 0.05) and *Cladosporium* (r^2^ = 0.071, p < 0.05) showed positive correlations with vegetation cover. Soil properties were also associated with fungal abundance: *Iodophanus* (r^2^ = 0.105, p < 0.01) and *Ascobolus* (r^2^ = 0.094, p < 0.01) were positively correlated with calcium, whereas *Rhizopus* (r^2^ = 0.060, p < 0.05) and *Filobasidium* (r^2^ = 0.086, p < 0.01) correlated with potassium and soil pH. Other genera (e.g., *Nothophoma*, *Tulostoma*, *Sporormia*, *Penicillium*, *Thanatephorus*, *Westerdykella*, *Aspergillus*, *Preussia*, *Pseudoarthrographis*, and *Saccobolus*) exhibited weak or non-significant associations (p > 0.05), highlighting the complexity of fungal-environment relationships within this ecosystem.

## Discussion

### Taxonomic structure and ecological roles of Saudi soil fungi

Our study reveals substantial regional variation in the mycobiome of Saudi Arabian desert soils, identifying over 14,600 OTUs. Earlier surveys from desert habitats in the region reported markedly fewer OTUs (e.g., 497–831; Moussa et al. 2017; Murgia et al. 2019; Alhuthali et al. 2024) and fewer species in pyrosequencing-based assessments (e.g., 60 fungal species along the Red Sea shoreline in Jeddah), underscoring the dependence of sequencing technology, marker length, and sampling intensity. The higher OTU richness observed in our study is consistent with PacBio long-read sequencing of full-length ITS region, which improves taxonomic resolution, combined with extensive standardized sampling that increases the detection of rare taxa. Across sites in Saudi Arabian desert, fungal communities were dominated by Ascomycota (>75% of fungal reads; 8,145 OTUs), mirroring global patterns in drylands where Ascomycota prevail due to resilience to nutrient scarcity, drought, and UV exposure (Maestre et al. 2015; Egidi et al. 2019; Cowan et al. 2022). Protective pigments such as melanin are likely to contribute to survival under harsh desert conditions (Challacombe et al. 2019). Dominant genera such as *Nothophoma* and *Pleiochaeta*, alongside *Alternaria* and *Fusarium*, reflect a mixture of saprotrophic and plant-associated/pathogenic strategies typical of arid soils (Rodriguez-Sanchez et al. 2021; Yiallouris et al. 2024). Other ecologically important genera (e.g., *Westerdykella*, known for its saprobic role in decomposing plant organic material, and *Ascobolus*, primarily a dung-inhabiting saprotroph) contribute to organic matter decomposition in soil ecosystems (Ebead et al. 2012; Sun et al. 2024). The presence of stress-tolerant taxa such as the basidiomycete *Naganishia* (halotolerant and psychrotolerant) further highlights the adaptive strategies employed by fungi in extreme environments.

In Saudi Arabian desert soils, saprotrophic fungi were the most abundant functional group, accounting for > 50% of total reads and comprising 4,668 OTUs. These fungi, especially soil and litter saprotrophs, are crucial for breaking down organic matter and supporting nutrient cycling and soil stability key processes in arid ecosystems (Baldrian 2016; Li et al. 2019; Kang et al. 2023). Different types of saprotrophs, particularly wood and dung decomposers, further enhance soil organic matter and nutrient availability. This high prevalence aligns with global studies from other dry habitats, where saprotrophs are similarly significant. Pathotrophic fungi (mainly plant pathogens) formed the next largest guild (27% of reads), with higher abundances at sites with moderate vegetation and strong environmental stress (e.g., Giya, Al Zulfi, and Wadi Liyyah), consistent with reports of increased pathogen abundance under intermediate stress in drylands (Maestre et al. 2015; Li et al. 2016; Zhang et al. 2023b). Although mutualistic fungi such as arbuscular mycorrhizal fungi (AMF) and ectomycorrhizal fungi (EcMF) represented only 1.4% of the community in this study, they can be functionally important for plant adaptation to water scarcity and nutrient-poor soils (Harris-Valle et al. 2018; Muhammad et al. 2024). The dominance of Glomeraceae among AMF may reflect efficient plant water and nutrient uptake under drought stress (Madouh and Quoreshi 2023), whereas the truffle-forming EcMF family Pezizaceae may contribute to soil stability and ecosystem resilience in desert regions (Ferreira et al. 2023). Overall, the composition of mycorrhizal guilds is consistent with functional adaptation to extreme environmental stressors rather than arbitrary distribution (Tedersoo et al. 2020).

### Alpha diversity patterns across arid soil environments

OTU richness, Shannon diversity, and Pielou’s evenness varied strongly across sites. OTU richness and Shannon diversity were lowest in the hyper-arid central and southern desert interiors and higher in the northern and northwestern regions, which generally have higher elevation and greater vegetation cover. In contrast, evenness tended to be higher in hyper-arid sites and slightly lower in more diverse, vegetated sites. This does not necessarily indicate stronger dominance in the latter communities. Rather, it is consistent with an increase in low-abundance taxa as environmental conditions become less restrictive and habitat heterogeneity increases. Because Pielou’s evenness is normalized by the maximum possible Shannon diversity for a given richness (i.e., ln(S)), the addition of many rare taxa can reduce evenness, even without an increase in dominance. Thus, the pattern likely reflects a shift from strongly filtered, species-poor assemblages to richer communities with a longer tail of rare taxa. This interpretation is qualitatively consistent with classical rank-abundance expectations (i.e., from more geometric-series-like assemblages under strong stress toward longer-tailed distributions in richer communities) (Maestre et al. 2015; Egidi et al. 2019; Grishkan et al. 2021). The patterns of soil fungal alpha diversity observed across Saudi Arabian deserts closely parallel global trends found in other arid and high-altitude sites. In the hyper-arid central and southern interior regions (Riyadh, Al Zulfi, Alwasel, and Razor Bay), both OTU richness and Shannon diversity showed synchronous spatial patterns, with low taxonomic richness associated with lower Shannon values. This indicates that environmental stress in hyper-arid regions causes a simultaneous decline in both species count and community evenness. The findings suggest that aridity serves as a uniform filter, affecting structural balance and taxonomic variety. Minor variations between the indices highlight subtle fluctuations in evenness, yet both metrics confirm a significant reduction in alpha diversity in hyper-arid regions. This reflects the strong limiting effects of aridity and minimal vegetation, much like in the Namib and Atacama Deserts (Maestre et al. 2015; Araya et al. 2020; Vikram et al. 2023). Only a limited set of resilient fungal taxa can persist, resulting in communities frequently dominated by the same adaptable species across multiple sites. In contrast, moister habitats support greater ecological differentiation, with unique taxa and more intricate community structures emerging under those more favourable environmental conditions. Overall, these trends highlight the strong influence of aridity, vegetation, and altitude on fungal diversity patterns in Saudi Arabia, echoing patterns observed in drylands worldwide (Wang et al. 2015; Neilson et al. 2017).

Our study demonstrated that vegetation coverage is a significant factor shaping fungal alpha diversity in desert ecosystems. Sites with higher vegetation cover, such as those in the northwestern escarpments, southwestern highlands (Khushaym, Al Khomrah, Fayfa, and Darb Bani Sha’ba), and irrigated oases (Riyadh: Najd Oasis), showed greater richness and Shannon diversity. In contrast, sites with little vegetation, including Al Khaldiyah, Alwasel, Al Muthallath, Green Duba, and Hafirat Al-Aida, were characterized by low fungal diversity. These dry, sparsely vegetated coastal plains and hyper-arid desert interiors supported only stress-tolerant taxa. Similar patterns are reported from other drylands. For example, in the Israeli desert, thermotolerant melanized species dominated less vegetated areas, while more diverse, less stress-tolerant communities thrived in vegetated sites (Grishkan 2018, 2019). The Atacama Desert also illustrated that vegetation enhances fungal diversity by providing favourable microsites and nutrient enrichment (Fuentes et al. 2020). In the Namib Desert, vegetated soils showed stable fungal communities, unlike barren soils which exhibited instability (Ramond et al. 2014). Furthermore, in China’s Mu Us Desert, revegetation altered fungal compositions towards more functionally diverse groups (Zhang et al. 2021). Overall, these studies underscore the critical role of vegetation in sustaining fungal diversity in arid environments.

Fungal evenness was also associated with vegetation indices (notably LSWI), elevation, and vegetation cover. LSWI reflects vegetation moisture and biomass and thus serves as a proxy for surface conditions relevant to soil fungi. Importantly, remote-sensing indices are predictions rather than direct mechanistic drivers and they summarize vegetation and moisture patterns that can covary with microsite availability, litter inputs, and soil water status (Chen et al. 2025; Zhang et al. 2025). Overall, vegetation indices, elevation, and pH emerged as consistent predictors of fungal alpha diversity in desert regions (Shen et al. 2020; Chen et al. 2023; Egidi et al. 2023; Zhang et al. 2025).

Elevational differences significantly impacted fungal diversity in Saudi Arabia, with high-altitude locations such as Jabal Dakah, Mt. Lawz, Maksanah, Bani Aamer, and Zahir supporting greater alpha diversity than low-elevation arid and coastal zones. This trend aligns with studies from other arid environments such as Atacama (Santiago et al. 2018), Namib (Scola et al. 2018; Vikram et al. 2023), and Negev (Grishkan et al. 2021), suggesting that cooler, wetter highlands provide more favorable soil moisture and organic inputs that support diverse fungal communities. Conversely, low-lying areas face drought and salinity stress, favoring extremotolerant fungi (Alotaibi et al. 2020).

Soil pH also emerged as a significant but context-dependent factor, with lower values closer to neutrality supported higher fungal richness, whereas highly alkaline soils constrained diversity. This pattern agrees with global and dryland studies showing diversity peaks at near neutral to mildly alkaline pH but declines under extreme alkalinity (Orlando et al. 2012; Tedersoo et al. 2014; Arifuzzaman et al. 2016; Grishkan 2018; Scola et al. 2018; Vasar et al. 2021; Fuentes et al. 2022).

The severe conditions of Saudi deserts, characterized by high temperatures, low moisture, and scarce organic matter, result in comparatively low overall microbial and fungal diversity when contrasted with temperate ecosystems (Maestre et al. 2015; Khan and Khan 2020). Nevertheless, the soil itself is a critical factor, defining the boundaries of life by harboring specialized, stress-tolerant microbial communities essential for nutrient cycling, soil stability, and ecosystem resilience (Egidi et al. 2019). Recent research from the Arabian Peninsula illustrates this principle, revealing that fungal communities are not uniformly distributed; diversity is the highest in localized niches with more favourable conditions, such as soils with greater vegetation cover, higher organic matter content, or access to intermittent water sources, while being the scarcest in the most arid and nutrient-poor areas (Murgia et al. 2019; Alrajhi et al. 2024). A distinguishing feature of Saudi deserts, relative to some global and regional analogs, is the degree to which environmental gradients reduce the community to a few adaptable or specialized genera. While other deserts may retain moderate levels of functional redundancy, Saudi arid soils often show sharper taxonomic filtering, particularly in the most alkaline or unvegetated sites. However, the underlying ecological processes remain consistent: across deserts worldwide, gradients of plant structure, soil chemistry, and climatic variability interact to determine which fungal lineages persist, with communities shaped by both environmental stress and the availability of key microhabitats (Scola et al. 2018; Egidi et al. 2019; Liu et al. 2021; Naidoo et al. 2022).

### Fungal beta diversity in arid ecosystems and its environmental structuring

Patterns of community dissimilarity in Saudi Arabian desert soils indicate that a set of key environmental variables is associated with fungal community structuring among sites. Multivariate analyses (PERMANOVA and NMDS) based on abundance-weighted Bray–Curtis dissimilarity highlighted that soil pH, elevation, vegetation indices, vegetation cover, and to a lesser extent nutrients such as calcium and potassium as significant correlates of compositional difference across sites. These findings are consistent with work from the Namib and Atacama deserts, where local edaphic variation and climate stress structure fungal communities (Scola et al. 2018; Naidoo et al. 2022).

In arid environments, such as the Atacama, Namib, Negev, and Mu Us deserts, differences in elevation and vegetation influence fungal community structure. In the Atacama Desert, hyper-arid lowlands and highlands exhibit varying fungal diversity (Knief et al. 2020). The Namib Desert’s fairy circles support distinct plant types, affecting microbial communities (Van der Walt et al. 2016; Vikram et al. 2023). Higher altitudes in the Negev Desert foster diverse fungal populations linked to vegetation, contrasting with barren soil conditions (Grishkan and Temina 2023). Similarly, the Mu Us desert’s fungal diversity has increased due to vegetation recovery (Zhang et al. 2021). On a larger scale, elevation enhances beta diversity through abiotic factors like temperature and moisture, while vegetation acts as a biotic filter, promoting species-specific fungal communities (Prober et al. 2015; Navarro-Noya et al. 2021; Peng et al. 2022; Mikryukov et al. 2023).

Our study indicated that soil pH has a significant impact on the fungal beta diversity in Saudi Arabian soils, serving as an essential environmental filter that affects community turnover. While the variation attributed to pH was smaller in comparison to vegetation and elevation, its impact on fungal composition shows that alkaline conditions limit diversity and foster communities dominated by stress-tolerant species. This finding aligns with other desert ecosystems: in the Namib Desert, pH along with other soil parameters influenced dune fungal communities (Johnson et al. 2017; Vikram et al. 2023); in the Atacama Desert, extreme alkalinity restricted fungal diversity and promoted extremotolerant groups (Scola et al. 2018); and in the Negev Desert, pH gradients significantly shaped fungal community differentiation in sparsely vegetated areas (Grishkan 2018). Global studies show that pH is one of the most reliable indicators of fungal β diversity, with changes in acidity or alkalinity causing community differences through environmental filtering and resource availability (Rousk et al. 2010; Tedersoo et al. 2014; Huang et al. 2024). Fungi have broader pH tolerance than bacteria, yet their community turnover is responsive to fluctuations at both acidic and alkaline extremes. This shows that vegetation and elevation enhance beta diversity in dry soils, but soil pH consistently influences fungal community formation both in deserts and on a global scale.

Fungal community turnover in desert soils is notably associated with soil nutrient levels such as potassium, phosphate, and calcium, indicating that minor changes in nutrient availability can lead to compositional changes. Evidence from deserts like the Namib and Atacama suggests that environmental factors, including variable nutrient availability, shape fungal community differentiation (Johnson et al. 2017; Guevara-Araya et al. 2022; Vikram et al. 2023). Variability in carbon, nitrogen, and phosphorus enhances niche differentiation and beta diversity (Nemergut et al. 2013; Johnson et al. 2017; Vikram et al. 2023). Specifically, soil phosphorus and carbonate levels in the Namib desert define xeric zones where distinct assemblages exist, influenced by rainfall (Scola et al. 2018). In the Atacama Desert, fungal diversity is affected by edaphic factors along aridity gradients (Guevara-Araya et al. 2022). Restoration studies in the Mu Us Desert reveal that plants improve soil organic matter and nitrogen, altering fungal communities (Zhang et al. 2021). While global studies argue that soil nitrogen impacts fungal turnover, nutrient effects on biodiversity patterns may be limited at larger scales (Tedersoo et al. 2022; Mikryukov et al. 2023). Nevertheless, nutrient amendments have been shown to significantly enhance beta diversity, emphasizing the contextual importance of nutrient availability in shaping fungal community composition (Zhou et al. 2020). These ecological dynamics are clearly reflected in the strategies of different fungal genera. Saudi sites with higher vegetation consistently support a wider range of fungal taxa, including *Alternaria*, *Nothophoma*, *Pleiochaeta*, *Cladosporium*, and *Aspergillus*, which are frequently reported in arid soils globally (Tedersoo et al. 2014; Grishkan 2018; Egidi et al. 2019; Murgia et al. 2019). Genera such as *Fusarium* and *Penicillium*—both cosmopolitan and highly versatile—are common across Saudi and other desert soils, benefiting from organic inputs and able to thrive as saprotrophs or endophytes (Egidi et al. 2019; Guevara-Araya et al. 2022; Isola and Prenafeta-Boldú 2025). The persistence of stress-tolerant and melanin-rich genera such as *Westerdykella* and *Exophiala* (Grishkan 2018; Scola et al. 2018; Egidi et al. 2019), as well as lichenized forms such as *Endocarpon* and *Verrucaria* (Liu et al. 2021), further underscores the importance of plant-fungal interactions and habitat heterogeneity for community assembly (Egidi et al. 2019; Yu et al. 2022). However, compared to some less extreme deserts, Saudi soils often exhibit sharper taxonomic filtering, suggesting that only the most competitive or stress-adapted lineages maintain stable populations under persistent environmental constraints (Scola et al. 2018; Naidoo et al. 2022). Ultimately, these taxonomic patterns are the biological manifestation of the strong environmental filtering exerted by the desert biome.

### Environmental gradients and the structural complexity of desert fungal communities

Fungal communities in Saudi Arabian deserts are strongly shaped by environmental gradients that act as filters on diversity. Ascomycota dominate across all sites, while Basidiomycota and non-Dikarya groups are less common. This taxonomic structure is typical of arid regions where water stress, salinity, and alkaline soils limit fungal groups to those with high stress tolerance (Tedersoo et al. 2014; Egidi et al. 2019). In Saudi soils, the effect is more substantial than in some other deserts, with communities reduced to a few melanized and extremotolerant taxa, much like patterns reported from the Atacama and Namib (Grishkan 2018; Scola et al. 2018; Murgia et al. 2019). Structural complexity is the lowest in hyper-arid interiors where a few dominant genera prevail, while higher elevations and vegetated zones support more diverse assemblages (Egidi et al. 2019; Yu et al. 2022). Environmental filtering shifts communities toward stress-tolerant saprotrophs and lichenized fungi, while specialized symbionts are rare. This reduction in functional redundancy means that desert fungi, though less diverse, play critical roles in nutrient cycling, soil stability, and ecosystem resilience (Naidoo et al. 2022).

## Conclusion

This research highlights the substantial role of environmental factors in shaping the richness, diversity, and composition of soil fungal communities in Saudi Arabian soils. DNA metabarcoding corroborates and expands patterns identified in global drylands, where fungal communities are shaped by strong environmental filtering that favors stress-tolerant taxa under chronic resource limitation (Tedersoo et al. 2014; Scola et al. 2018; Egidi et al. 2019; Yu et al. 2022). Saudi Arabian soils were dominated by Ascomycota and Basidiomycota, while multiple additional phyla were present at lower abundance. Soil pH, elevation, vegetation indices, and vegetation cover were key predictors of fungal diversity and community composition. The positive association between fungal diversity and vegetation cover highlights the importance of vegetation-mediated microhabitats in sustaining fungal life in arid landscapes, consistent with patterns reported worldwide (Maestre et al. 2015; Wang et al. 2015; Neilson et al. 2017; Egidi et al. 2019; Yu et al. 2022). Together, these findings support the view that desert fungi combine stress tolerance with ecological specialization, contributing to nutrient cycling and ecosystem functioning in harsh environments.

## Additional information

### Use of AI

No use of AI was reported.

### Author ORCIDs

Israel Mani https://orcid.org/0000-0001-5777-7804

Vladimir Mikryukov https://orcid.org/0000-0003-2786-2690

Saad Alkahtani https://orcid.org/0000-0001-7381-5110

Leho Tedersoo https://orcid.org/0000-0002-1635-1249

### Data availability

All the data that support the findings of this study are available in the main text or Supplementary Information. DNA sequences recovered in this study were deposited in the European Nucleotide Archive (ENA) under accession numbers ERR15816935 - ERR15817019 (Project ID PRJEB102166, Study ID ERP183567).

## Supplementary materials

Supplementary material 1 Supplementary information Data type docx https://binary.pensoft.net/file/1565001This dataset is made available under the Open Database License (http://opendatacommons.org/licenses/odbl/1.0/). The Open Database License (ODbL) is a license agreement intended to allow users to freely share, modify, and use this Dataset while maintaining this same freedom for others, provided that the original source and author(s) are credited. Israel Mani, Vladimir Mikryukov, Saad Alkahtani, Leho Tedersoo
