# Supplementary material for: Desert mycobiome of Saudi Arabia is driven by vegetation patterns
Source: MycoKeys. 2026 Mar 19;130:71–100. doi: 10.3897/mycokeys.130.176937 (PMC13022707; doi:10.3897/mycokeys.130.176937)
Supplement: Supplementary material 1 — Supplementary information [file mycokeys-130-071-s001.docx]

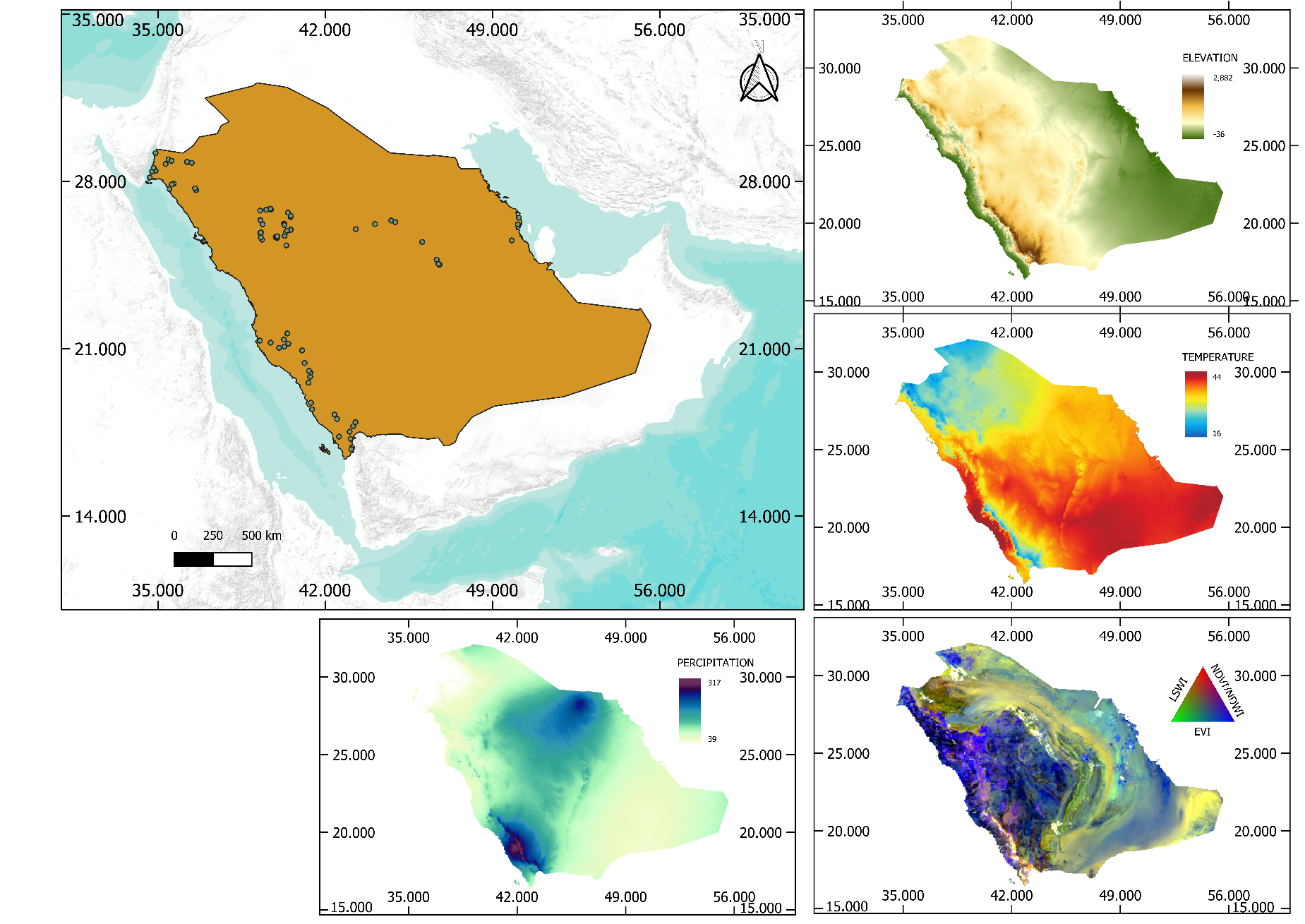


**Supplementary Figure S1:** Geographic distribution of sampling sites and major environmental gradients across Saudi Arabia. The central map highlights sampling locations, while the accompanying panels illustrate variations in elevation, temperature, precipitation, and vegetation indices (LSWI, NDVI, NDWI, and EVI). Together, these layers reveal the strong environmental heterogeneity shaping the country’s diverse ecological and climatic pattern Maps were generated using QGIS (QGIS Development Team 2024).

**Supplementary Table S1:** The table summarizes the statistical relationships between environmental factors and fungal alpha diversity, highlighting how these variables influence diversity measures and identifying the most significant predictors across the study sites.

|  | Estimate | Std. error | t-value | p-value | Adj. R² |
| --- | --- | --- | --- | --- | --- |
| **OTU richness** |  |  |  |  |  |
| Plant_coverage | 83 | 16.06 | 5.17 | <0.001 *** | 0.226 |
| Soil_ph | -909 | 327.25 | -2.78 | 0.007 ** | 0.18 |
| Elevation | 66.11 | 13.39 | 4.94 | <0.001 *** | 0.095 |
|  |  |  |  |  |  |
| **Shannon index** |  |  |  |  |  |
| Plant_coverage | 0.3 | 0.05 | 5.6 | <0.001 *** | 0.178 |
| Soil pH | -3.19 | 1.09 | -2.94 | 0.004 ** | 0.173 |
| Elevation | 0.26 | 0.04 | 5.81 | <0.001 *** | 0.152 |
|  |  |  |  |  |  |
| **Evenness** |  |  |  |  |  |
| Elevation | -0.158 | 0.049 | -2.31 | 0.021 * | 0.091 |
| Vegetation coverage | -0.117 | 0.042 | -2.77 | 0.006 ** | 0.072 |
| LSWI vegetation index | 1.59 | 0.672 | 2.37 | 0.018 * | 0.05 |

Reference

QGIS Development Team (2024) QGIS Geographic Information System. Version 3.40.2-Bratislava. QGIS Association. https://www.qgis.org
